# Relationship between occupational stress and sleep quality among emergency nurses

**DOI:** 10.3389/fpubh.2025.1699441

**Published:** 2025-12-03

**Authors:** Yizhu Gao, Xiaofang Ma, Yusi Wang, Hao Zhang, Luying Zhong, Zhenfei Yuan, Xiaoli Chen

**Affiliations:** 1 Department of Emergency Medicine, West China Hospital, Sichuan University, Chengdu, China; 2 West China School of Nursing, Sichuan University, Chengdu, China; 3 Disaster Medical Center, Sichuan University, Chengdu, China; 4 Nursing Key Laboratory of Sichuan Province, Chengdu, China

**Keywords:** emergency nurses, occupational stress, sleep disorders, night shifts, workload, effort-reward imbalance, overcommitment

## Abstract

**Background:**

Emergency nurses face high occupational stress and long working hours, which may contribute to sleep disorders. However, the extent and nature of this association remain unclear.

**Objective:**

This study assessed the relationship between occupational stress and sleep disorders among emergency nurses and identified their contributing factors.

**Study design:**

A stratified cluster sampling method was employed between 26 December 2023 and 18 January 2024, based on the seven geographical regions of China (Northeast, North, East, Central, South, Southwest, and Northwest China). Emergency nurses aged ≥18 years, with ≥1 year of emergency care experience, and no psychiatric disorder history were included. Nurses undergoing advanced training or those on sick leave, maternity leave, or breastfeeding leave for ≥1 month were excluded. Participants completed a structured questionnaire including demographic data, an occupational stress assessment using a five-point Likert scale from 1 to 5, and sleep disorder quality evaluation. Logistic regression analysis was conducted to identify factors associated with sleep disorders, whereas Spearman’s correlation analysis evaluated the relationship between occupational stress and sleep disorders.

**Results:**

A total of 1,551 questionnaires were collected. After excluding 11 invalid responses, 1,540 were analyzed. Binary logistic regression identified key risk factors for sleep disorders, including 11–15 years of work experience (OR = 1.692), weekly working hours of 49–58 h (OR = 1.784) or ≥59 h (OR = 2.268), night shift frequency, and overcommitment scores (OR = 1.098). A significant positive correlation was found between occupational stress and sleep disturbances (*p* < 0.05).

**Conclusion:**

These findings highlight the need for hospital administrators to implement targeted interventions, such as psychological support programs, shift rotation optimization, and stress management training. Future research should focus on longitudinal designs to establish causal pathways and evaluate the effectiveness of interventions aimed at improving sleep quality among emergency nurses.

## 1 Introduction

Sleep disorders encompass a range of functional impairments in the sleep–wake cycle, typically characterized by prolonged sleep latency, difficulties in maintaining sleep, and early morning awakenings. Daytime manifestations commonly include fatigue, lack of concentration, impaired cognitive function, irritability, anxiety, and low mood. A cross-sectional study conducted in the United States revealed that 77.4% of nurses experienced suboptimal sleep quality, with 7.4% suffering from severe insomnia (1). Similarly, in Chinese public hospitals, the prevalence of poor sleep quality and severe sleep problems among emergency nurses was reported to be 65.8 and 61.4%, respectively (2). Inadequate sleep can result in concentration deficits, memory decline, increased absenteeism, higher risk of injuries, and diminished job performance. Furthermore, poor sleep quality is associated with adverse health outcomes, including elevated risks of dementia, cardiovascular diseases, and all-cause mortality. Notably, nurses with sleep difficulties are 15 times more likely to screen positive for depression risk (3).

Occupational stress, often termed “job stress” or “work pressure,” refers to the process wherein workplace stressors elicit psychological, behavioral, or physiological stress responses that may lead to long-term health consequences. This phenomenon affects various sectors involving both physical and mental labor. A UK-based study identified nursing as one of the three most stressful occupational groups (4). In recent years, occupational stress has gained increasing scholarly attention. Epidemiological data indicate that the prevalence of occupational stress among nurses is 38.8% in Europe, 49.0% in North America, 42.7% in South America, and as high as 57.4% in Asia. In China, over two-thirds (68.7%) of emergency nurses report experiencing occupational stress (5). Contributing factors to occupational stress can provoke a spectrum of stress-related responses, including depression, anxiety, and insomnia.

A substantial body of research has documented associations between occupational stress and various physical and mental health conditions, reduced work performance, absenteeism, high staff turnover, and decreased job satisfaction (6–8). A study focusing on police officers highlighted a bidirectional relationship between sleep and stress, demonstrating that chronic occupational stress exposure significantly increases the likelihood of sleep disturbances (9). Correspondingly, a cross-sectional investigation into psychosocial work stress and health risks found that emergency department nurses experiencing occupational stress and overcommitment faced elevated risks of developing stress-related diseases later in life (10). As a high-risk population for occupational stress, nurses frequently encounter persistent sleep problems that not only compromise their physical and mental well-being but also jeopardize medical safety, thereby posing substantial threats to patient care quality. Nevertheless, the specific correlation between occupational stress and sleep disturbances among emergency nurses remains inadequately elucidated, warranting further investigation.

Therefore, this study aimed to assess the relationship between occupational stress and sleep disorders among emergency nurses and to identify contributing factors. We hypothesized that higher occupational stress, longer working hours, and frequent night shifts would be significantly associated with poorer sleep quality.

## 2 Materials and methods

### 2.1 Study participants

From December 2023 to January 2024, a stratified cluster sampling method was employed based on geographical regions of China. Inclusion Criteria were as follows: (1) Emergency nurses aged ≥18 years, (2) Possessing at least one year of experience in emergency care, (3) No prior history of diagnosed psychiatric disorders. Exclusion Criteria was nurses on sick leave, maternity leave, or breastfeeding leave for one month or more. The sample size was determined using the formula for cross-sectional studies, the prevalence of sleep disorders among nurses in the previous studies were estimated at 59.9–77.4%, with a margin of error of 5% and a confidence level of 95%, the calculated sample size ranged from 269 to 370, with the maximum sample size of 370 selected.

### 2.2 Research instruments

General Demographic Information Collection Form: This form collected details such as gender, age, educational background, years of work experience, night shift status, income level, and marital status.

#### 2.2.1 Occupational stress questionnaire

The effort-reward imbalance questionnaire, developed by the German scholar Siegrist and adapted into Chinese version by scholar Dai (11), was used to assess occupational stress levels among emergency nurses. This questionnaire comprises 6 items on effort, 11 items on reward, and 5 items on overcommitment, with each item rated on a five-point Likert scale from 1 to 5. Higher scores indicate greater levels of occupational stress. In this study, the Cronbach’s *α* coefficient for this scalescale was 0.88, three dimensions are 0.894, 0.952, and 0.922, respectively.

#### 2.2.2 Sleep disorder survey

The self-administered sleep questionnaire (SSQ) (12) was used to evaluate sleep disorders among emergency department nurses. This questionnaire assesses 3 sleep symptoms, time to fall asleep, sleep continuity, and early morning awakening, through 3 items. Sleep disorder was defined as meeting at least one of the following: (1) taking ≥30 min to fall asleep; (2) unable to resume sleep after waking more than once a week; (3) early morning awakening with difficulty resuming sleep more than once a week. This composite definition was used to classify participants in Table 1. In this study, the Cronbach’s *α* coefficient for this scale was 0.809, showing good reliability and validity. Participants were instructed to report their overall sleep quality over the past month, considering both workdays and days off, to minimize bias from transient shift schedules.

**Table 1:** Analysis of sleep quality.

Variable		Frequency	Percentage (%)
Time to fall asleep	0–10 min	154	10.0
	11–30 min	507	32.9
	31–59 min	433	28.1
	1–2 h	305	19.8
	>2 h	141	9.2
Inability to resume sleep after waking	Never (or almost never)	256	16.6
	A few times a year	405	26.3
	More than once a month	379	24.6
	More than once a week	345	22.4
	Almost every night	155	10.1
Early morning awakening and difficulty resuming sleep	Never (or almost never)	280	18.2
	A few times a year	372	24.2
	More than once a month	340	22.1
	More than once a week	356	23.1
	Almost every night	192	12.5
Sleep disorders	Present	913	59.3
	Absent	627	40.7

### 2.3 Data collection

An electronic version of the survey questionnaire was created using the Wenjuanxing platform. To ensure data authenticity and consistency across multiple centers, the following measures were implemented: (1) All questionnaire items were set as mandatory to avoid missing data; (2) Each IP address was restricted to one response to prevent multiple submissions by the same individual; (3) Coordinators from each center received unified training to standardize survey administration. Key concepts and instructions related to the survey were standardised through training to ensure that the designated hospital personnel responsible for administering the survey provided accurate and consistent explanations of its content. The QR code for the survey was distributed via the WeChat platform. Participants were required to read and sign the informed consent form before completing the questionnaire. The questionnaires were eliminated, such as identical responses.

### 2.4 Statistical analysis methods

Statistical analyses were conducted using SPSS version 26.0. Descriptive statistics were performed for demographic data. Categorical variables were expressed as frequencies (n) and percentages, while continuous variables were presented as means and standard deviations. The Mann–Whitney U test was used for continuous variables, and the Chi-square test was used for categorical variables in univariate analysis. For variables with more than two categories in univariate analysis, the Chi-square test was applied with the binary sleep disorder outcome. Binary logistic regression analysis was employed to explore factors influencing sleep disorders, and Spearman’s rank correlation analysis was used to examine the relationship between occupational stress and sleep disorders. Potential confounding variables (e.g., age, gender, years of experience, night shift) were included in the binary logistic regression model to control for their effects. Multicollinearity was assessed using Variance Inflation Factor (VIF), with all values below 10 indicating no significant multicollinearity.

### 2.5 Ethics approval statement

This study was approved by the Ethics Review Committee of West China Hospital, Sichuan University (approval no. 2024.309). Written informed consent was obtained from all participants prior to data collection.

## 3 Results

### 3.1 Descriptive analysis of general demographic information of study participants

During the study period, a total of 1,551 questionnaires were collected. After excluding 11 questionnaires with identical responses, 1,540 were included in the final analysis. The participants were recruited from seven geographical regions of China. The regional distribution was as follows: North China (9%), Northeast China (15.3%), East China (21.6%), Central China (9%), South China (8.2%), Southwest China (16.9%), and Northwest China (19.7%). The basic characteristics of the participants were as follows: 1,211 were female, accounting for 78.6%; 1,354 had an education level of a bachelor’s degree or higher, representing 87.9%; 960 worked more than 40 h per week, accounting for 62.5%; and 1,359 had night shifts, representing 88.2%. See Table 2 for details.

**Table 2:** General demographic information (*n* = 1,540).

Variable	Category	Frequency	Percentage (%)
Age group	20–29 years	582	37.8
	30–39 years	734	47.7
	40–49 years	183	11.9
	≥ 50 years	41	2.7
Gender	Female	1,211	78.6
	Male	329	21.4
Marital status	Single	560	36.4
	Married	980	63.6
Parental status	No children	710	46.1
	Has children	830	53.9
Education level	Associate degree	186	12.1
	Bachelor’s degree or higher	1,354	87.9
Professional title	Junior	907	58.9
	Intermediate	574	37.3
	Senior	59	3.8
Years of work experience	≤5 years	491	31.9
	6–10 years	493	32.0
	11–15 years	300	19.5
	16–20 years	121	7.8
	> 20 years	135	8.8
Weekly working hours	<40 h	577	37.5
	41-48 h	780	50.6
	49-58 h	123	8.0
	<59 h	60	3.9
Night shift status	No	181	11.8
	Yes	1,359	88.2
Night shift frequency	0 times/month	181	11.8
	1–4 times/month	239	15.5
	5–8 times/month	659	42.8
	≥9 times/month	461	29.9
Monthly income	<4,000 RMB/month	57	3.7
	4,000–5,999 RMB/month	149	9.7
	6,000–7,999 RMB/month	250	16.2
	8,000–9,999 RMB/month	368	23.9
	≥10,000 RMB/month	716	46.5
Smoking status	Non-smoker	1,453	94.4
	Smoker	87	5.6
Drinking status	Non-drinker	1,355	88.0
	Drinker	185	12.0

### 3.2 Descriptive analysis of survey results on occupational stress and sleep quality

Regarding occupational stress, the total score was 55.55 ± 16.78, with an effort score of 19.22 ± 5.35, a reward score of 23.7 ± 9.10, and an overcommitment score of 12.60 ± 4.74. In terms of sleep quality, 59.3% of nurses experienced sleep disorders. Among them, 57.1% of nurses took 30 min or more to fall asleep, and 16.6% of nurses reported never being able to fall back asleep after waking up during the night. For detailed information, please refer to Tables 1, 3.

**Table 3:** Scores of occupational stress scores.

Variable	Dimension	Min	Max	Scores (x̅ ± s)
Occupational stress	Effort	6	30	19.22 ± 5.35
	Reward	11	55	42.28 ± 9.10
	Overcommitment	5	25	12.60 ± 4.74
	Total score	22	110	55.55 ± 16.78

### 3.3 Analysis of factors influencing sleep disorders

To examine the factors associated with sleep disorders, sleep disorders were used as the dependent variable, while general demographic characteristics and occupational stress were used as independent variables.

#### 3.3.1 Univariate screening

The univariate analysis revealed that years of work experience, weekly working hours, night shifts, and occupational stress were significantly associated with sleep disorders among emergency nurses (*p* < 0.05). See Table 4 for details.

**Table 4:** Results of univariate screening for sleep disorders.

Variable	Category	Sleep disorders	Normal sleep	χ^2^/t	*P*
		*N* = 913	*N* = 627		
Age group	20–29 years	321	261	12.539	**0.006**
	30–39 years	456	278		
	40–49 years	118	65		
	≥ 50 years	18	23		
Gender	Female	727	484	1.312	0.252
	Male	186	143		
Marital status	Single	327	233	0.291	0.590
	Married	586	394		
Parental status	No children	412	298	0.863	0.376
	Has children	501	329		
Education level	Associate degree	109	77	0.041	0.874
	Bachelor’s degree or higher	804	550		
Professional title	Junior	538	369	1.900	0.387
	Intermediate	345	229		
	Senior	30	29		
Years of work experience	≤5 years	263	228	15.428	**0.004**
	6–10 years	296	197		
	11–15 years	196	104		
	16–20 years	82	39		
	> 20 years	76	59		
Weekly working hours	<40 h	305	272	26.682	**0.000**
	41–48 h	473	307		
	49–58 h	89	34		
	<59 h	46	14		
Night shift frequency	0 times/month	90	91	14.386	**0.002**
	1–4 times/month	131	108		
	5–8 times/month	394	265		
	≥ 9 times/month	298	163		
Monthly income	<4,000 RMB/month	37	20	8.423	0.077
	4,000–5,999 RMB/month	93	56		
	6,000–7,999 RMB/month	164	86		
	8,000–9,999 RMB/month	217	151		
	≥10,000 RMB/month	402	314		
Smoking status	Non-smoker	855	598	2.081	0.177
	Smoker	58	29		
Drinking status	Non-drinker	803	552	0.003	0.959
	Drinker	110	75		
Occupational stress	Effort	19.97 ± 5.13	18.14 ± 5.47	6.708	**0.000**
	Reward	25.04 ± 9.23	21.81 ± 8.56	6.939	**0.000**
	Overcommitment	13.49 ± 4.77	11.30 ± 4.38	9.147	**0.000**
	Total score	58.50 ± 16.67	51.25 ± 16.01	8.522	**0.000**

The bold values mean the *P* < 0.05.

#### 3.3.2 Results of multivariate analysis

The results of the binary logistic regression analysis indicated that years of work experience (11–15 years), weekly working hours (49–58 h and ≥59 h), night shift frequency, and overcommitment scores were significant predictors of sleep disorders among emergency nurses. Specifically, emergency nurses with 11–15 years of work experience had a 1.692 times higher likelihood of developing sleep disorders compared to those with less than 5 years of work experience. Additionally, for each one-unit increase in the overcommitment score, the risk of sleep disorders increased by 1.098 times. See Table 5 for details.

**Table 5:** Results of multivariate binary logistic regression analysis on sleep disorders.

Variable		B	Standard deviation	*P*	Exp(B)	95% CI for exp(B)	
						Lower limit	Upper limit
Age group	Control: 20–29 years				1		
	30–39 years	−0.12	0.221	0.587	0.887	0.575	1.368
	40–49 years	0.02	0.393	0.959	1.02	0.472	2.204
	≥ 50 years	−0.522	0.558	0.349	0.593	0.199	1.771
Years of work experience	Control: ≤5 years				1		
	6–10 years	0.285	0.217	0.189	1.33	0.869	2.035
	11–15 years	0.526	0.268	0.049	1.692	1.002	2.859
	16–20 years	0.607	0.366	0.098	1.834	0.895	3.76
	> 20 years	0.43	0.459	0.349	1.537	0.625	3.779
Weekly working hours	Control: <40 h				1		
	41-48 h	0.203	0.116	0.080	1.225	0.976	1.537
	49-58 h	0.579	0.228	0.011	1.784	1.141	2.788
	<59 h	0.819	0.331	0.013	2.268	1.186	4.336
Night shift frequency	Control: 0 times/month				1		
	1–4 times/month	0.44	0.221	0.046	1.553	1.008	2.394
	5–8 times/month	0.598	0.197	0.002	1.818	1.235	2.675
	≥9 times/month	0.654	0.206	0.002	1.923	1.283	2.881
Occupational stress	Effort score	0.007	0.013	0.595	1.007	0.981	1.034
	Reward score	−0.004	0.01	0.708	0.996	0.978	1.015
	Overcommitment score	0.094	0.021	0.000	1.098	1.054	1.144
Constant		−1.732	0.289	0.000	0.177		

#### 3.3.3 Results on the correlation between occupational stress and sleep disorders

A positive correlation was observed between occupational stress and sleep disorders among emergency nurses (*p* < 0.05). See Table 6 for details.

**Table 6:** Correlation between occupational stress and sleep disorders.

Variable			Effort	Reward	Overcommitment	Total score	Time to fall asleep	Sleep continuity and early morning awakening	Early morning awakening and difficulty resuming sleep	Total score
Occupational stress	Effort	Correlation coefficient	1							
	Reward	Correlation coefficient	0.501**	1						
	Overcommitment	Correlation coefficient	0.662**	0.759**	1					
	Total score	Correlation coefficient	0.777**	0.916**	0.905**	1				
Sleep disorders	Time to fall asleep	Correlation coefficient	0.175**	0.197**	0.254**	0.234**	1			
	Sleep continuity and early morning awakening	Correlation coefficient	0.342**	0.366**	0.454**	0.436**	0.489**	1		
	Early morning awakening and difficulty resuming sleep	Correlation coefficient	0.333**	0.339**	0.419**	0.408**	0.409**	0.839**	1	
	Total score	Correlation coefficient	0.337**	0.357**	0.446**	0.427**	0.725**	0.922**	0.896**	1

** indicate statistical significance at the 0.01 level (2-tailed).

### 3.4 Collinearity diagnosis

The collinearity diagnosis showed that all tolerance statistics were greater than 0.1 and all Variance Inflation Factor (VIF) values were less than 10, indicating no evidence of multicollinearity among the independent variables (Table 7).

**Table 7:** The result of collinearity diagnosis.

Variable		Linear correlation statistics	
		Tolerance statistics	VIF
	Constant		
Age group	Control: 20–29 years		
	30–39 years	0.238	4.208
	40–49 years	0.190	5.254
	≥ 50 years	0.370	2.705
Years of work experience	Control: ≤5 years		
	6–10 years	0.282	3.547
	11–15 years	0.259	3.854
	16–20 years	0.314	3.180
	> 20 years	0.180	5.566
Weekly working hours	Control: <40 h		
	41–48 h	0.839	1.192
	49–58 h	0.860	1.162
	<59 h	0.903	1.108
Night shift frequency	Control: 0 times/month		
	1–4 times/month	0.443	2.260
	5–8 times/month	0.301	3.320
	≥9 times/month	0.324	3.087
Occupational stress	Effort score	0.549	1.822
	Reward score	0.415	2.408
	Overcommitment score	0.314	3.185

## 4 Discussion

Emergency nurses serve as a vital force within the healthcare system, bearing the responsibility of treating critically ill patients. The nature of their work often entails 24-h availability, and the high-intensity, high-pressure environment presents significant challenges to their physical and mental well-being, among which sleep disorders are particularly prominent. This survey found that 59.3% of emergency nurses experienced sleep disorders, a finding consistent with the cohort study by Zhang et al. (13), which reported a prevalence of 59.9% among nurses. Adequate sleep is a physiological necessity, comparable to the need for food, and is crucial for sustaining life, health, and workplace safety (14–16). Good sleep quality is essential for nurses to deliver optimal patient care. Previous studies have similarly indicated a high prevalence of sleep disorders among nurses, with reported rates of 77.4% in the United States (1), 63.9% in China (17), and 57% in Taiwan (18), and a pooled prevalence of 61.0% according to Zeng et al. (19). These data suggest that sleep disorders are a common issue among nursing populations globally.

The results of the binary logistic regression analysis identified years of work experience (11–15 years), weekly working hours (49–58 h and ≥59 h), night shift frequency, and overcommitment scores as significant factors associated with the occurrence of sleep disorders among emergency nurses. Specifically, nurses with 11–15 years of experience had 1.692 times higher odds of sleep disorders compared to those with less than 5 years of experience. Moreover, for each one-unit increase in the overcommitment score, the risk of sleep disorders increased by 1.098 times. Due to prolonged exposure to high-pressure emergency settings, they may face dual pressures from career advancement and familial responsibilities, potentially leading to the accumulation of chronic stress, which could adversely affect sleep quality.

Furthermore, long-term shift work has been linked to reduced sensitivity of melatonin receptors, which may exacerbate sleep problems of emergency nurses. Extended weekly working hours (49–58 h or ≥59 h) were associated with more severe sleep disturbances. Such prolonged work hours may lead to persistent activation of the sympathetic nervous system, potentially impairing cellular repair processes and sleep efficiency. Frequent night shifts disrupt the body’s natural circadian rhythm, leading to dysregulation of the biological clock, which in turn may negatively impact sleep quality. Over time, such persistent disruptions are likely to contribute to ongoing sleep disturbances, with further implications for physical and mental health of emergency nurses.

This study also demonstrated a significant positive correlation between occupational stress and sleep disorders among emergency nurses (*p < 0.05). As crucial members of the healthcare system, emergency nurses operate under high-intensity pressure and complex working conditions. They are required not only to manage critically ill patients but also to respond to various emergencies and unpredictable situations. Within this demanding context, occupational stress among emergency nurses has become increasingly evident, with components such as effort, reward, and overcommitment significantly associated with their well-being. Emergency nurses often invest considerable effort into their work without receiving commensurate rewards. This effort-reward imbalance may contribute to feelings of frustration and dissatisfaction, which could further increase the risk of sleep disorders. Additionally, the high levels of concentration and dedication required in their roles often necessitate substantial overcommitment. While this may improve work efficiency and quality, it may also result in physical and mental exhaustion. Prolonged exposure to such demanding conditions appears to heighten the likelihood of developing sleep disturbances and other stress-related health issues. These findings suggest the need for targeted interventions to reduce occupational stress and improve sleep among emergency nurses, such as, optimizing shift schedules to limit consecutive nights; providing stress management and mindfulness training.

## 5 Limitations

It is important to note that this study demonstrates a correlation between occupational stress and sleep disorders, not causation. The cross-sectional design limits causal inference. The study participants were primarily emergency department nurses, making it necessary to further assess the generalizability of the findings to other hospital settings and clinical departments. Future research should consider conducting large-sample longitudinal cohort studies to elucidate the causal relationship between occupational stress and sleep disorders.

## 6 Conclusion

Years of work experience, weekly working hours, night shifts, and occupational stress are significant risk factors contributing to sleep disorders among emergency nurses. This study highlights a correlation between occupational stress and sleep disturbances in this population, with findings suggesting that greater occupational stress is associated with increased severity of sleep disorders.

## Data Availability

The raw data supporting the conclusions of this article will be made available by the authors, without undue reservation.
